# The effectiveness of cognitive- behavioral therapy on quality of life in women with hypothyroidism in the reproductive age: a randomized controlled trial

**DOI:** 10.1186/s13044-020-00080-z

**Published:** 2020-05-20

**Authors:** Sohaila Rezaei, Parvin Abedi, Elham Maraghi, Najmeh Hamid, Homaira Rashidi

**Affiliations:** 1 MS. c Candidate in Midwifery Department, Reproductive Health Promotion Research Centre, Ahvaz Jundishapur University of Medical Sciences, Ahvaz, Iran; 2 PhD in Community Nutrition, Associate Professor in Midwifery Department, Menopause Andropause Research Centre, Ahvaz Jundishapur University of Medical Sciences, Ahvaz, Iran; 3 PhD of Biostatistics, Assistant Professor, Department of Biostatistics and Epidemiology, Faculty of Public Health, Ahvaz Jundishapur University of Medical Sciences, Ahvaz, Iran; 4 Associate Professor of Clinical Psychology, Shahid Chamran University of Ahvaz, Ahvaz, Iran; 5 Associate Professor in Endocrinology Department, Diabetes Research Center, Health Research Institute, Ahvaz Jundishapur University of Medical Sciences, Ahvaz, Iran

**Keywords:** Cognitive behavioral therapy, Hypothyroidism, Quality of life

## Abstract

**Background:**

Worldwide, hypothyroidism affects 3.7% of the population, and is associated with impaired quality of life. This study aimed to evaluate the effect of cognitive- behavioral therapy (CBT) on the quality of life in women with hypothyroidism.

**Methods:**

96 women with hypothyroidism randomly allocated into two groups: CBT group (*n* = 48) and control group (n = 48). Women in the CBT group were classified into four sub-groups of 12, and each sub-group received eight sessions of counseling (each session lasting 90 min). We collected data using a demographic questionnaire and the 36-Item Short Form Health Survey (SF 36) for measuring the quality of life. We used the independent t-test, chi-square test and ANCOVA to analyze the data.

**Results:**

Five women from each group withdrew from the study, leaving 43 women in each group. The scores on physical functioning, physical health problems, social functioning and pain improved in the CBT group after the intervention, but the differences between the two groups were not significant. The scores on emotional health, emotional health problems, energy and emotions, and general health were significantly better in the CBT group than those in the control group (*p* < 0.05).

**Conclusion:**

Counseling using CBT can improve some aspects of quality of life, including emotional health, emotional health problems, energy and general health in patients with hypothyroidism.

**Trial registration number:**

Iranian Registry for Clinical Trials: 20190323043101 N1. https://www.irct.ir/

## Background

Hypothyroidism defined as the insufficient amount of thyroid hormones, that may result from disorders of the thyroid gland, pituitary gland or hypothalamus [1]. It is estimated it affects 3.7% of the population worldwide [2]. A cohort study (2006–2011) in Iran showed that the prevalence of hypothyroidism was 3.3 and 2.1 per 1000 (person-year) in adult women and men, respectively [3]. The Iranian government started to fortify the salt with iodine from 1990, and at the present time more than 95% of Iranian households use iodized salt, therefore, the risk of the goitre due to iodine insufficiency has decreased significantly [4].

The most commonly used treatment for hypothyroidism is levothyroxine. A study showed that women, who were diagnosed with hypothyroidism and underwent treatment with levothyroxine, showed improvement in the overall quality of life after six months follow up; however, some domains of the quality of life remained poor in comparison to healthy women [5]. In a study of 9491 Western European participants, Klaver et al. showed that women with increased TSH had scores of quality of life similar to those in euthyroid women [6]. In contrary, Shivaprasad et al., in their study of 244 women with hypothyroidism who were treated with thyroid hormone, found that despite the treatment, women had lower scores in six out of eight domains of quality of life [7]. Rakhshan et al. found that the spiritual health score of women with hypothyroidism was significantly lower than that in healthy women [8].

There are several methods to improve the quality of life in women with subclinical or clinical hypothyroidism. Werneck et al. found that a 16-week aerobic exercise could significantly improve different domains of quality of life, including physical functioning, general health, emotional health, mental, and physical components of quality of life [9].

Cognitive- behavioral therapy (CBT) is one of the counseling methods that can help people to change their negative thoughts [10]. The use of CBT could improve the quality of life in patients with cardiac disease [11], hepatitis B [12], and women with breast cancer [13]. To the best of our knowledge, no previous study has evaluated the effect of CBT on quality of life in women with hypothyroidism. Therefore, this study aimed to evaluate the effect of CBT on the quality of life of women who were under medication for hypothyroidism.

## Methods

This was a randomized controlled trial involving 96 women with hypothyroidism in reproductive age. This study started in May 2019 and completed in September 2019. Women with the following criteria were recruited in this study: age 18–45 years, who had basic literacy, hypothyroidism diagnosed with laboratory tests and confirmed by an endocrinologist, women who acquired score less than 60 of the total score of quality of life questionnaire (SF36) and on medication for hypothyroidism. Women with the following criteria were excluded from the study: pregnancy, thyroid malignancy, mental illness such as depression and dementia, any chronic diseases such as coronary heart disease, smoking and drug abuse, history of stressful life events during the past six months. The protocol of this study was approved by the Ethics Committee of Ahvaz Jundishapur University of Medical Sciences (Ref No: 1397.968). This study was also registered in the Iranian Randomized Controlled Trials (IRCT) registry, (20,190,323,043,101 N1).

The following formula was used to calculate the sample size [14]:

$$ n=\frac{{\left({Z}_{1-\frac{\alpha }{2}}+{Z}_{1-\beta}\right)}^2\left({s_1}^2+{s}_2^2\right)}{{\left(\mathrm{d}\right)}^2}=\frac{{\left(2.58+1.29\right)}^2\left({11.96}^2+{11.9}^2\right)}{(10)^2} $$

The power of the study was set at 90% and α = 0.01, S2 = S1 = 11.9 and d = 10. The sample size was calculated to be 48 in each group.

### Measures

In this study, a demographic questionnaire and the quality of life questionnaire (SF 36) was used to collect the data. The demographic questionnaire consisted of questions about age, age of spouse, employment, women and their husband’s educational attainment, economic status, any special diet, level of physical activity, duration of the disease, and obstetric history.

The SF36 questionnaire was designed by Ware and Sherbourne [15] and has 36 questions about the different domains of physical functioning (10 questions), role limitations due to the physical health problems (4 questions), bodily pain (2 questions), general health perception (6 questions), social functioning (2 questions), vitality (4 questions), mental health (5 questions), and role limitations due to emotional problems (3 questions). Questionnaire scores range from zero to 100, with a score of zero indicating the worst health status and a score of 100 indicating the best health status. This questionnaire was validated to use in Iran by Montazeri et al. in 2006 [16].

### Randomization

Eligible women who provided consent were randomly assigned into two groups of CBT and control. We used block randomization with a block size of 4 and an allocation ratio of 1:1. For allocation concealment, each participant received a code and these codes were kept in the opaque sealed envelopes by the secretary of the clinic until the commencement of the intervention.

### Intervention

The CBT group received eight sessions (a session per week) of counseling based on cognitive -behavioral therapy and the control group did not receive any intervention. The counseling session was held in a group of 12 and each session lasted 90 min. In the first session, participants introduced themselves, and one of the researchers (SR) explained about hypothyroidism, and how this disease can influence the life and activities of patients. The second session consisted of how women can reduce stress, according to the CBT. In the third session, after reviewing the homework, women received information about the incorrect thoughts and attitudes about hypothyroidism, and the relationship between negative thoughts and health. In the fourth session, participants received information about the reconstruction and change of irrational and negative attitudes and attributes and ineffective schemas about it. In this session, homework considered in the practice of muscle relaxation, verbal and nonverbal communication that enhance creativity and mastery over life and how to adopt with events and irrational beliefs about events.

In the fifth session, participants were trained about strengthening positive self- talk and visualizing successful relationships, cognitive challenges, and enhancing realism. In the sixth session, the researcher introduced muscle relaxation training for each muscle group individually, informing participants about stress, physical stress and how to increase awareness about physical symptoms of stress (mental-physical exercise training and imaging exercise). Participants also learned about diaphragmatic breathing exercises, how to identify negative thoughts and correct perceptions of stressful situations and introduce a thought assessment process. In the seventh session, participants learned about self-practicing for heat and heaviness, identifying logical and irrational self-talk, challenging negative self-talk, reconstruction and changing irrelevant negative thoughts, and demonstration of the effects of revised thinking and practicing reasoning thoughts. In the 8th session, participants received instructions about the logic of self-learning with visualization, positive self-induction, meditation, introducing effective coping steps and practice and finally the researcher summarized all sessions.

For ethical consideration, the control group received one compact disk on behavioral therapy at the end of the intervention. The blinding of the researcher and participants was not possible in this study. Although there was no blinding in this study, the researchers and participants did not know who was in which group until the intervention was started. Also, the statistician was not aware of the grouping.

### Statistical analyses

We entered all data in the SPPS version 22. The normal distribution of data was assessed using the Shapiro-Wilk test. The independent t-test was used for testing numerical data between the two groups, while the chi-square test was used for comparing categorical data. For assessing variables that were measured twice, the ANCOVA was used for comparison. *P* < 0.05 was considered significant.

## Results

**Fig. 1:**
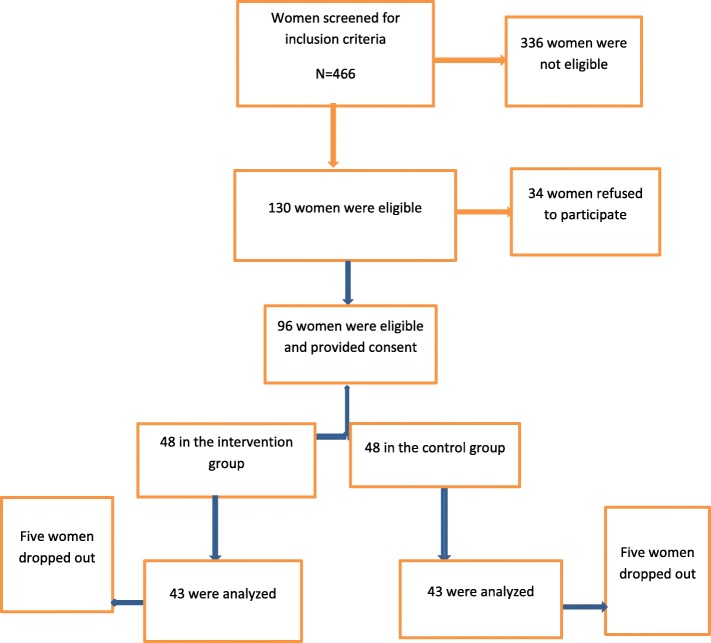
Recruitment and retention of participants in the study

Forty-eight women were recruited in each group; however, five women in each group withdrew from the study because of personal reasons. The recruitment and retention of participants are shown in Fig. 1. The age of participants in the intervention group was significantly higher than that in the control group (35 ± 6.03 vs. 32.5 ± 7.42). Except for the age, the two groups did not show any significant difference in terms of body mass index, duration of disease, marital status, educational level of women and their spouses, employment and economic status (Table 1).

**Table 1:** Socio-demographic characteristics of participants in the two groups

Variables	CBT *N* = 43	Control N = 43
	Mean ± SD	
Age (y)	35.6 ± 6.03	32.5 ± 7.42
Body mass index (kg/m^2^)	28.44 ± 4.9	27.56 ± 5.37
Duration of illness (y)	7.23 ± 7.05	4.95 ± 4.05
	N (%)	
**Marital status**		
Single	5 (11.6)	10 (23.3)
Married	37 (86)	33 (76.7)
Divorces	1 (2.3)	0
**Educational level**		
High school	6 (14)	10 (23.3)
Diploma	19 (44.2)	9 (20.9)
University education	18 (41.9)	24 (55.8)
**Spouse’s education**		
High school	7 (18.9)	7 (21.2)
Diploma	11 (29.7)	13 (39.4)
University education	19 (51.4)	13 (39.4)
**Employment**		
Housewife	30 (69.8)	33 (75.7)
Employee	13 (30.2)	10 (23.3)
**Economic status**		
Poor	5 (11.6)	10 (23.3)
Moderate	31 (72.1)	30 (69.8)
Good	7 (16.3)	3 (7)

CBT: cognitive- behavioral therapy

P for all variable was not significant except for age

Table 2 shows some general and obstetric characteristics of participants. Before the diagnosis of their disease, 44.2 and 34.9% of participants in the intervention group and the control group had physical activity, respectively. However, only 16.3% of women in the two groups had physical activity at the time of the study. Only a few women in the two groups received education or counseling about their disease (14 and 16.3% in the intervention and control groups, respectively).

**Table 2:** General and obstetric characteristics of participants in the two groups

Variables	CBT *N* = 43	Control *N* = 43	*P* value
	N (%)		
Physical activity before the disease	19 (44.2)	15 (34.9)	0.25
Physical activity at the present time	7 (16.3)	7 (16.3)	0.61
Special diet	3 (7)	4 (9.3)	0.50
Received education about the disease	6 (14)	7 (16.3)	0.50
	Median (Range)		
Number of pregnancies	2.0 (0.0–6.0)	2.0 (0.0–5.0)	2.0 (0.0–6.0)
Number of deliveries	2.0 (0.0–3.0)	2.0 (1.0–3.0)	2.0 (0.0–3.0)
Number of children	2.0 (0.0–3.0)	2.0 (0.0–3.0)	2.0 (0.0–3.0)

Table 3 shows different domains of quality of life in two groups of CBT and control before and after the intervention. Although the physical functioning improved significantly in the CBT group after the intervention (from 59.6 ± 19.4 to 66.3 ± 21.8, *p* = 0.01), the difference between the CBT and control groups was not significant (*p* = 0.10). The two groups did not show any significant difference in physical health problems, social functioning and bodily pain after intervention.

**Table 3:** Different domains of quality of life in two groups of CBT and control before and after intervention

Variables	CBT N = 43		Control *N* = 43		P value between groups
	Mean ± SD				
	Before	After	Before	After	
**Physical functioning**	59.6 ± 19.4	66.3 ± 21.8	60.1 ± 16.4	60.9 ± 17.5	0.10
P value before-after within each group	0.01		0.66		
**Role limitations due to the physical health problems**	30.8 ± 23.6	51.1 ± 29.3	38.3 ± 21.3	42.4 ± 20.7	0.05
P value before-after within each group	< 0.0001		0.36		
**Emotional health**	42.2 ± 14.5	53.9 ± 16.2	41.4 ± 15.4	42.9 ± 14.6	0.003
P value before-after within each group	< 0.0001		0.49		
**Role limitations due to emotional problems**	18.6 ± 19.6	35.6 ± 32.03	17.05 ± 16.8	20.1 ± 19.4	0.01
P value before-after within each group	0.001		0.43		
**Vitality and emotions**	33.6 ± 13.1	48.1 ± 15.8	36.1 ± 14.4	37.6 ± 13.6	0.001
P value before-after within each group	< 0.0001		0.51		
**Social functioning**	52.3 ± 22.3	63.3 ± 21.8	54.3 ± 18.6	54.6 ± 20.7	0.11
P value before-after within each group	0.92		0.03		
**Bodily pain**	39.1 ± 14.06	57.3 ± 24.1	46.8 ± 19.3	51.1 ± 18.8	0.08
P value before-after within each group	< 0.0001		0.12		
**General Health**	30.6 ± 17.8	43.6 ± 18.4	29.3 ± 14.2	32.2 ± 17.3	0.004
P value before-after within each group	< 0.0001		0.26		

The scores of emotional health, emotional health problems, energy and emotions, and general health were significantly improved after the intervention in the CBT group as compared to those in the control group (*p* < 0.05). Overall physical health and overall spiritual health significantly improved in the CBT group as compared to the control group.

## Discussion

This study was designed to evaluate the effect of CBT on the quality of life in women with hypothyroidism. The results showed that, although the scores of physical functioning, role limitations due to the physical health problems, social functioning, bodily pain, improved significantly after the intervention in the CBT group, the differences between the two groups were not significant. Hypothyroidism is a disease manifested with the reduction of thyroid hormone and may accompany some neuropsychiatric symptoms such as cognitive function and mood. In severe hypothyroidism, some symptoms similar to melancholia and dementia may occur [17]. In this regard, Joffe et al. found that CBT could improve depression as well as thyroid hormone levels in patients with overt and subclinical hypothyroidism [18].

In the present study, we found that although the scores of physical functioning and physical health problems improved in the CBT group, the difference between the two groups was not significant. Although we could not find any study on the effect of CBT on the physical functioning of hypothyroidism, White et al. in a large randomized controlled trial found that the adjuvant therapy of exercise and CBT could significantly improve chronic fatigue syndrome when added to medical care [19]. These results are consistent with our results.

In the present study, the scores of emotional health, emotional health problems, energy and emotions, and general health, were significantly improved after eight sessions of CBT in the CBT group as compared to the control group. In line with our findings, Nekouei et al. found that CBT could significantly improve the quality of life in patients with cardiovascular disease [11]. In a review, Lukkahatai et al. suggested that CBT is more likely to improve the psychological aspects of chronic illnesses than the physical and biological aspects [20]. Finally, Castro et al. randomized 93 patients with chronic pain into two groups of CBT and control, and found that CBT significantly improved depression, physical limitation, the general state of health and limitation by emotional aspects [21].

### Strengths and limitation of the study

This is the first study to evaluate the effect of CBT on the quality of life in women with hypothyroidism. The results of this study, however, should be considered with caution in light of few limitations. First, we did not check the level of thyroid hormone before and after the intervention. Second, the responses to the questions of quality of life questionnaire may be affected by recall bias. Third, we only followed participants for eight weeks, further studies with longer follow-up are recommended. Fourth, we used block randomization with non-variable block size that may lead to predicting intervention. Lacking of blinding is another limitation. Although there was no blinding in this study, the researchers and participants did not know who was in which group until the intervention. And finally, because the SF36 questionnaire was completed by participants in the clinic and the researcher was there to resolve any ambiguity, it may have affected their responses.

## Conclusion

This study showed that intervention with CBT can improve some aspects of quality of life, including emotional health, emotional health problems, energy and general health in a patient with hypothyroidism. Further studies with longer follow up are recommended.

## Data Availability

Data of this study will be available upon the request from the corresponding author.

## Abbreviation

CBT: Cognitive- behavioral therapy
TSH: Thyroid stimulating hormone
